# Evaluation of altered brain activity in type 2 diabetes using various indices of brain function: A resting-state functional magnetic resonance imaging study

**DOI:** 10.3389/fnhum.2022.1032264

**Published:** 2023-01-09

**Authors:** Ge Zhang, Taiyuan Liu, Wei Wei, Rui Zhang, Huilin Wang, Meiyun Wang

**Affiliations:** ^1^Department of Radiology, Henan Provincial People's Hospital, Zhengzhou, China; ^2^Department of Radiology, Bethune International Peace Hospital, Shijiazhuang, China; ^3^Laboratory of Brian Science and Brain-Like Intelligence Technology, Institute for Integrated Medical Science and Engineering, Henan Academy of Sciences, Zhengzhou, China

**Keywords:** amplitude of low-frequency fluctuation (ALFF), regional homogeneity (ReHo), voxel-mirrored homotopic connectivity (VMHC), resting-state functional magnetic resonance imaging (fMRI), type 2 diabetes mellitus

## Abstract

**Background:**

Type 2 diabetes mellitus (T2DM) has been identified as a risk factor that increases the rate of cognitive decline. Previous studies showed that patients with T2DM had brain function alterations based on a single index of resting-state functional magnetic resonance imaging (rs-fMRI). The present study aimed to explore spontaneous brain activity in patients with T2DM by comparing various rs-fMRI indices, and to determine the relationship between these changes and cognitive dysfunction.

**Methods:**

A total of 52 patients with T2DM and age- and sex-matched control participants were included in this study. The amplitude of low-frequency fluctuation (ALFF), regional homogeneity (ReHo), and voxel-mirrored homotopic connectivity (VMHC) values were calculated to represent the status of spontaneous neural activity. The Montreal Cognitive Assessment (MoCA) was used for the rapid evaluation of cognition in all subjects. Pearson correlation and mediation analyses were conducted to investigate the relationship between rs-fMRI indices and clinical parameters such as fasting glucose, disease duration, and MoCA.

**Results:**

Patients with T2DM had alterations of concordant spontaneous brain activity in brain areas including the bilateral cerebellum posterior lobe, the left inferior temporal gyrus (ITG.L), the parahippocampal gyrus, and the left supplementary motor area (SMA.L). The indices were significantly correlated to each other in most of the detected brain areas. Positive correlations were observed between fasting glucose and neural activity in the surrounding areas of the left insula and the inferior frontal gyrus. MoCA scores were negatively correlated with the ReHo values extracted from the left anterior occipital lobe and the superior cerebellar cortex and were positively correlated with VMHC values extracted from the left caudate and the precentral gyrus (PreCG). No significant mediation effect of abnormal brain activity was found in the relationship between clinical parameters and MoCA scores.

**Conclusion:**

The current study demonstrated the functional concordance of abnormal brain activities in patients with T2DM by comparing ALFF, ReHo, and VMHC measurements. Widespread abnormalities mainly involved in motor and sensory processing functions may provide insight into examining T2DM-related neurological pathophysiology.

## Introduction

Type 2 diabetes mellitus (T2DM) has been consistently associated with a variety of complications that can affect cognition and increase the risk of dementia (Cheng et al., 2012). In patients with T2DM, cognitive decline mainly includes memory decline, reduced information processing speed, learning deficits, and other symptoms that can be used as the progressive clinical hallmark of dementia (Biessels et al., 2006, 2014; Kivipelto et al., 2018; Kellar and Craft, 2020). Individuals with T2DM have been linked to a 1.5-fold increase in the development of mild cognitive impairment (Luchsinger, 2012). An epidemiological study demonstrated a 65% increase in the risk of developing Alzheimer's disease (AD) in patients with T2DM (Arvanitakis et al., 2004). While mild cognitive impairment is an independent cause of dementia with conversion rates of 50%, the co-occurrence of diabetes will further increase the risk (Velayudhan et al., 2010; Xu et al., 2010). Notably, large longitudinal population-based studies showed that the rate of cognitive decline is accelerated in elderly people with T2DM (Hassing et al., 2004). Several hypotheses have been proposed to illustrate the precise mechanisms underlying T2DM-related cognitive dysfunction, including neurogenesis in the hippocampus, the breakdown of the blood–brain barrier, and the toxic effects of high glucose concentration on neurons (Umegaki, 2014). However, the exact neuropathophysiological mechanism of T2DM-induced cognitive impairment is still unknown.

The neuroimaging method has proven to be a very useful tool to investigate T2DM-induced brain abnormalities (Biessels and Reijmer, 2014). White matter lesions, cerebral atrophy, and lacunar infarcts are commonly reported and are assumed to correlate with cognitive dysfunction (van Agtmaal et al., 2018; Zhuo et al., 2019; Xiong et al., 2022). Disordered concentrations of brain metabolites were detected using magnetic resonance spectroscopy in patients with T2DM and were also reported to be related to cognitive impairment (d'Almeida et al., 2020; Yang et al., 2020). However, neural activity cannot be monitored using these methods, which would make it a sensitive biomarker ahead of a brain lesion (Jones, 2012; Yao et al., 2021). Task-based functional magnetic resonance imaging (task-based fMRI) was utilized to couple neural activation and cognition-related task performance for patients with T2DM (Duarte et al., 2015; He et al., 2015). Findings from these task-based fMRI studies suggest that T2DM may alter the neural process underlying cognitive function. However, the heterogeneity of the results and the inconvenience of conducting the experiment limit the further application of task-based fMRI to neuroimaging studies in the diabetic brain.

Resting-state functional magnetic resonance imaging (rs-fMRI) has become a promising approach to evaluating spontaneous neural activity (Barkhof et al., 2014). Researchers calculated functional connectivity (FC) to investigate functional changes and found alterations in the neural functional networks in patients with T2DM (Musen et al., 2012; Chen et al., 2014; Zhang et al., 2015; Liu et al., 2019). However, the FC method focuses on the synchronization between distinct brain regions and requires an *a priori* setting of the seed region to perform the connectivity calculation. The rs-fMRI metrics provide an alternative way to reflect brain activity. Amplitude of low-frequency fluctuation (ALFF) and regional homogeneity (ReHo) analyses are the two commonly used indices to depict neural activity in rs-fMRI data (Liu et al., 2013; Xue et al., 2022). ALFF directly characterizes the intensity of spontaneous neural activity in each voxel, whereas ReHo estimates the functional synchronization of a given voxel and its neighbors (Zang et al., 2004, 2007).

Using ALFF and ReHo, abnormalities of regional spontaneous neural activity in patients with T2DM have been widely reported and produced various results. Several of the brain regions showing decreased or increased ALFF/ReHo were reported while integrating existing studies. For example, Xia et al. (2013) demonstrated that patients with T2DM had abnormal neural activity in the temporal lobe, the fusiform gyrus, and the occipital lobe, and Liao et al. (2019) observed abnormal neural activity in the anterior cingulate gyrus, the cuneus, the precuneus, and the middle frontal gyrus. Meanwhile, inconsistent results were reported in brain regions. For instance, there was increased (Wang et al., 2014, 2019; Zhou et al., 2014; Liu et al., 2020) as well as decreased (Peng et al., 2016; Wang et al., 2017, 2019; Liao et al., 2019) regional activity in the cerebellum, the fusiform, the cingulate cortex, and the precuneus. Although comorbidities and small samples may partly explain these findings, the central question is how is a neural function manifested in T2DM. A previous study indicated that ReHo and ALFF might be complementary to each other to measure global spontaneous brain activity (An et al., 2013). Afterward, Cui et al. (2014) observed the coexistence of functional intensity and the coherence of abnormalities in several brain regions and considered that the results represented reliable information. Therefore, a combination of various rs-fMRI indices will provide more information that will help to indicate the intrinsic brain activity alterations in T2DM (Yao et al., 2021).

Voxel-mirrored homotopic connectivity (VMHC) provides a voxel-wise measurement for interhemispheric FC (Gee et al., 2011). It quantitatively evaluates functional homotopy by calculating the time series correlation between each voxel and its mirror in the contralateral hemisphere (Zuo et al., 2010; Kelly et al., 2011). Unlike the common FC analysis, the VMHC method does not require a prior specification of the brain area as a “seed-region” to perform the calculation. While the synchronization of spontaneous activity between the mirrored regions in each bilateral hemisphere is an important feature of brain function, studies demonstrated that VMHC is a useful indicator to investigate the relationship between brain dysfunction and neuropsychiatric diseases (Guo et al., 2013; Li et al., 2019) as well as T2DM (Wang et al., 2020; Zhang et al., 2021). Therefore, the VMHC method offers an FC-based analysis tool at the voxel level, indicating a possible joint analysis along with ALFF and ReHo.

In this study, ALFF, ReHo, and VMHC were applied together to investigate spontaneous neural activity in patients with T2DM. The goal of the present work is to provide a comprehensive understanding of spontaneous neural activity in patients with T2DM. We hypothesized that (1) more brain areas with abnormalities would be detected using ALFF, ReHo, and VMHC; (2) abnormal values of ALFF, ReHo, or VMHC would be detected within the specific brain regions; and (3) abnormal neural activity would be related to T2DM-related biometric parameters and cognitive states.

## Methods and materials

### Participants

All participants were recruited from the endocrinology department at the Henan Provincial People's Hospital and *via* advertisements between November 2019 and October 2021. T2DM was diagnosed according to the criteria published by the World Health Organization in 1999 (American Diabetes Association, 2018). Detailed inclusion criteria included a patient aged between 40 and 75 years, disease duration >1 year, routine treatment with hypoglycemic agents, and right-handedness. Control subjects were matched to patients with T2DM in terms of age, sex, handedness, and education. Participants with a history of hypoglycemic episodes, alcoholism, drug abuse, brain injury, or neurological or psychiatric diseases that could lead to cognitive impairment were excluded. Patients with hearing/visual difficulties, a rating score of white matter changes >1, or MR contraindications were also excluded. Control subjects were excluded if they had (1) a fasting blood glucose level >7.0 mmol/L; (2) a glucose level >7.8 mmol/L after an oral glucose tolerance test; or the (3) presence of cognitive impairment. Vascular risk factors were not listed as the exclusion criteria, which might help improve the generalizability of the study results.

This study was approved by the ethics committee of a local hospital, and written informed consent was obtained from each participant.

### MRI data acquisition

All participants received magnetic resonance imaging (MRI) examinations using a Siemens 3.0 T Prisma scanner (Erlangen, Germany) with a Standard Siemens 8-channel head coil at the Radiology Department of Henan Provincial People's Hospital. Sound-proof equipment like an earplug was used to protect subjects' hearing during the scan. All subjects in the scanner were instructed to keep still, awake, and their eyes closed. Structural images were acquired using the three-dimensional T1-weighted spoiled gradient recalled echo sequence. The scan parameters were as follows: 174 axial slices, repetition time (TR) = 1,900 ms, echo time (TE) = 2.52 ms, thickness = 1 mm, field of view (FOV) = 256 mm × 256 mm, matrix = 256 × 256, voxel size = 1 mm × 1 mm × 1 mm, and FA = 9°. Functional images were acquired using a gradient-echo planar imaging sequence. The scan parameters were as follows: 33 axial slices, TR = 2,000 ms, TE = 30 ms, FOV = 240 mm × 240 mm, matrix = 64 × 64, voxel size = 3.75 mm × 3.75 mm × 3.75 mm, and FA = 90°, 210 time points.

### Clinical and cognitive scale tests

Anthropometric and medical information was recorded from all participants, including age, gender, weight, height, the duration of diabetic disease, glycosylated hemoglobin (HbAlc), fasting plasma glucose (FPG), total cholesterol, triglycerides (TAG), high-density lipoproteins (HDL), low-density lipoproteins (LDL), systolic blood pressure (SBP), and diastolic blood pressure (DBP). The duration of diabetic disease was defined as the interval between the age at first diagnosis to an MRI scan. Body mass index (BMI) was calculated based on weight and height to measure body fat. Hypertension was defined as SBP > 160 mmHg, DBP > 95 mmHg, or a self-reported medication history of hypertensive drugs (van den Berg et al., 2010). Blood samples were collected after an overnight fast of at least 8 h to measure blood glucose along with HbA1c and cholesterol levels.

Cognitive function was assessed using the Montreal Cognitive Assessment (MoCA) for each subject (Nasreddine et al., 2005). All participants completed MoCA tests before the MRI scan. The MoCA scale attempts to quantitatively gauge cognitive function in 11 sections with a total of 30 possible points, which are, respectively, “alternating trail making,” “visuoconstructional skills (cube),” “visuoconstructional skills (clock),” “naming,” “memory,” “attention,” “sentence repetition,” “verbal fluency,” “abstract thinking,” “delayed recall,” and “orientation.” The test was performed in a quiet, private room by professional neurological physicians following standard procedures. It has been concluded that MoCA is a useful and sensitive means of detecting mild cognitive impairment. Any score equal to 26 or >26 is considered normal.

### Data preprocessing

Resting-state fMRI data preprocessing was carried out using the toolbox Data Processing & Analysis for Brain Imaging (DPABI) (Yan et al., 2016) throughout Statistical Parametric Mapping 12 in the Matlab 2016b environment, with the following steps: (1) the first 15 time points were removed to avoid initial signal fluctuations; (2) slice timing correction was achieved by temporally aligning all slices to a manually designated reference slice; (3) six head motion parameters were extracted and regressed to a state of controlling motion interference; (4) fMRI normalization was performed in three steps: first, a T1-weighted anatomical image was normalized to the T1 template in the standard Montreal Neurological Institute (MNI) space; second, a T1-weighted anatomical image was co-registered to the mean image of the time series; and third, fMRI was normalized to the MNI space and resampled to 3 mm × 3 mm × 3 mm; (5) a Gaussian kernel of 4-mm full width at half maximum was adopted to conduct spatial smoothing (Cui et al., 2014, 2015); (6) linear detrending and band-pass filtering (frequency of 0.01–0.08 Hz) were used to eliminate unwanted noise and baseline drift; and (7) multiple linear regression was performed to remove the effects of irrelevant covariates, including Friston 24 motion, the global mean signal, white matter signal, and cerebrospinal fluid signal.

### ALFF, ReHo, and VMHC analyses

Amplitude of low-frequency fluctuations was calculated based on preprocessed images. The time series of each voxel was processed using the Fourier transform algorithm (Zang et al., 2007). As the power of each frequency point was proportional to the signal amplitude in the time domain, the square root was extracted to represent the corresponding signal amplitude at the frequency point. Then, the averaged square root over 0.01–0.08 Hz in each voxel was taken as the raw ALFF (Jiang and Zuo, 2016). The mean ALFF was used for standardization purposes, and finally, the resulting voxel-wise ALFF value was obtained.

To perform the ReHo analysis, the smoothing procedure was skipped before completing the calculation. ReHo distribution maps were generated by computing the concordance of the Kendall coefficient of a given voxel with 26 neighboring voxels after detrending and band-pass filtering (Zang et al., 2004). *Z*-transformation was performed to standardize the ReHo value of each voxel. Finally, the resulting ReHo data were smoothed for further analysis.

The VMHC value was determined by computing the Pearson correlation coefficient of the fMRI signal between the mirrored voxels in contralateral hemispheres. For statistical comparison, the correlation values were Fisher *z*-transformed.

### Statistical analysis

Differences in demographics, clinical variables, and cognitive performance between groups were compared using SPSS software (version 22; SPSS, Chicago, IL, USA). An independent-sample *t*-test and the chi-squared test were performed for continuous variables and categorical variables, respectively. The normality test was conducted ahead of time to examine whether the data conformed to the normal distribution. *p*-values of < 0.05 were considered statistically significant.

For the *z*-value maps, the inter-group differences of ALFF, ReHo, and VMHC were investigated using voxel-based independent-sample *t*-tests *via* DPABI software, as described earlier. The Gaussian Random Field (GRF) method, a cluster-based approach, was used to perform a multi-comparison correction. A voxel threshold was needed to cut the number of independent tests from the voxel number down to the cluster number. Then, a cluster threshold was set as the criterion after a multi-comparison correction among clusters. A voxel-level threshold of a *p*-value of < 0.001 and a cluster-level threshold of a *p*-value of < 0.05 were recommended (Yan et al., 2009; Peng et al., 2016; Wang et al., 2017, 2019; Liao et al., 2019; Liu, P. H. et al., 2021).

### Correlation analysis

To explore the association among the abnormalities of regional rs-fMRI indices, bivariate correlation tests were performed between every two indices. Regions that show significant differences were set as regions of interest (ROIs). The mean ALFF, ReHo, and VMHC values for each ROI were extracted and correlated with one another. To investigate the relationship among ALFF/ReHo/VMHC values, clinical variables, and cognition, a correlation analysis was repeated based on ROIs. A multi-comparison correction was performed to determine the most significant correlations.

### Mediation analysis

Fasting plasma glucose and the duration of the disease are considered to be underlying risk factors for cognitive impairment in patients with T2DM. Given that abnormal brain activity was potentially associated with significant clinical parameters and MoCA scores, we further assessed whether abnormal brain activity mediated the association between FPG/disease duration and MoCA scores in patients with T2DM. The mediation analysis was performed using the PROCESS toolbox developed by Andrew F. Hayes, which was implemented in SPSS software (Hayes, 2012). FPG and disease duration acted as the independent variables, while the MoCA score was a dependent variable. The mediator was abnormal brain activity associated with clinical parameters. A simple mediation model (model = 4) with a 5,000-bootstrap sample for significance testing was applied to conduct the analysis.

## Results

### Demographic and clinical characteristics

A total of 104 participants (52 patients with T2DM and 52 control subjects) who met the criteria were finally enrolled for data analysis. Demographic and clinical characteristics are presented in Table 1. These two groups showed no difference in terms of age, sex, or BMI. Meanwhile, no significant statistical difference was observed in total blood lipid levels or blood pressure. Particularly, the MoCA score between the two groups showed less difference with a *p*-value of > 0.05, suggesting similar levels of cognition between patients with T2DM and control subjects.

**Table 1:** Demographic and clinical characteristics of participants.

**Characteristics**	**Patients with T2DM**	**Control subjects**	***p-*Value**
Age (years)	53.9 ± 9.5	53.0 ± 7.7	0.59
Sex (men/women)	21/31	29/23	0.12
Height (m)	1.68 ± 0.07	1.65 ± 0.08	0.10
Weight (kg)	72.37 ± 11.46	69.24 ± 11.13	0.14
BMI (kg/m^2^)	25.52 ± 3.11	25.06 ± 2.82	0.41
Disease duration (years)	9.1 ± 6.5		
HbAlc (%)	8.22 ± 1.7		
Fasting glucose (mmol/L)	9.20 ± 2.67		
Cholesterol (mmol/L)	4.38 ± 1.04	4.41 ± 0.74	0.87
Triglyceride (mmol/L)	2.13 ± 2.25	1.90 ± 1.24	0.47
High density lipoprotein (mmol/L)	1.23 ± 0.44	1.19 ± 0.33	0.64
Low density lipoprotein (mmol/L)	2.44 ± 0.85	2.71 ± 0.59	0.06
Arterial blood pressure			
Systolic blood pressure (mmHg)	120.3 ± 6.6	119.1 ± 5.6	0.28
Diastolic blood pressure (mmHg)	78.7 ± 5.1	77.4 ± 4.4	0.13
Cognitive performance			
MoCA	25.08 ± 2.10	25.73 ± 1.88	0.08

Data are presented as mean ± SD.

### ALFF, ReHo, and VMHC analyses

**Figure 1:**
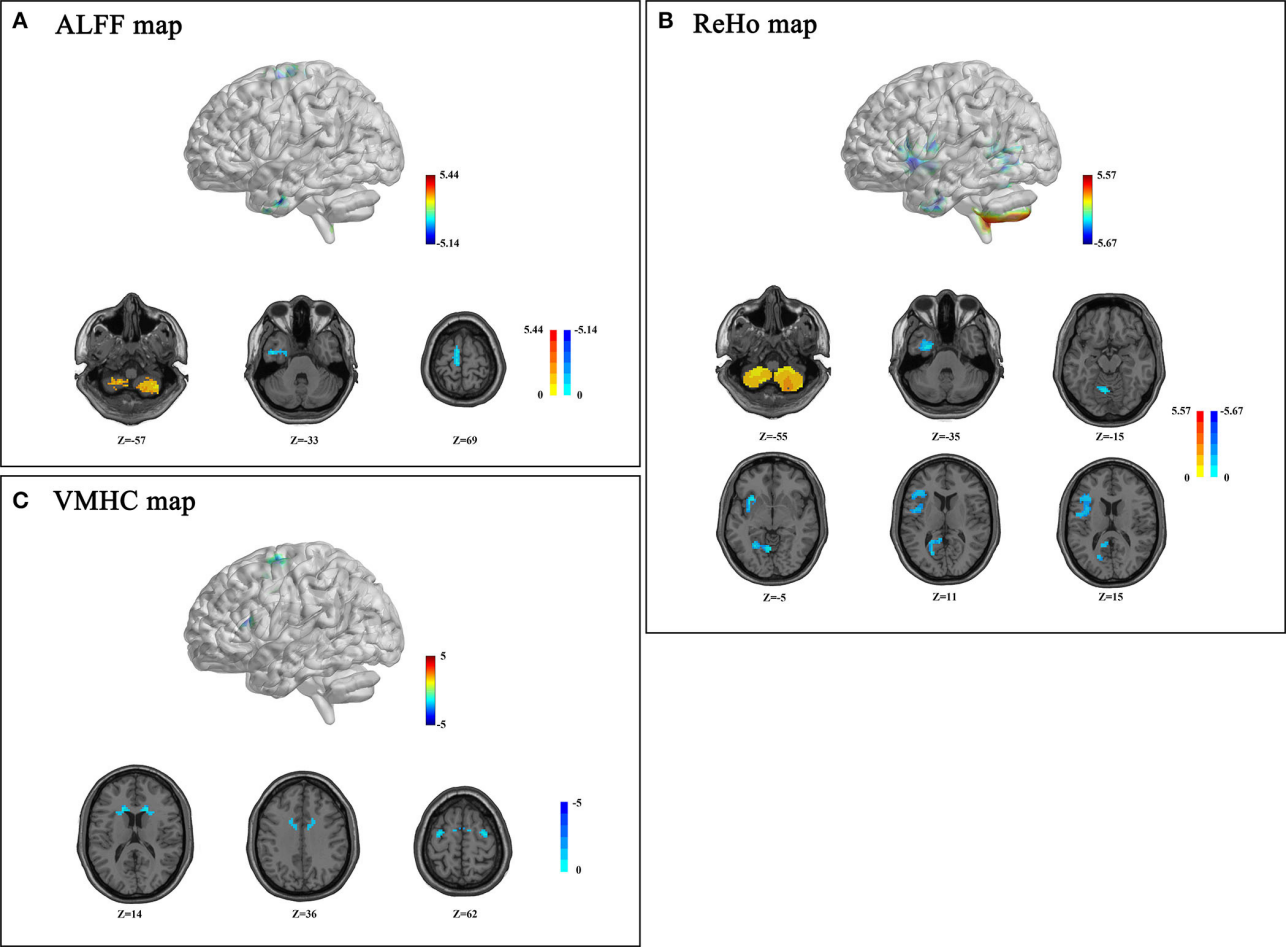
Amplitude of low-frequency fluctuation (ALFF) **(A)**, regional homogeneity (ReHo) **(B)**, and voxel-mirrored homotopic connectivity (VMHC) **(C)** differences between patients with type 2 diabetes mellitus (T2DM) and control subjects (*p* < 0.05, Gaussian Random Field (GRF) corrected). **(A)** Patients with T2DM presented decreased ALFF values in the bilateral inferior cerebellum, left inferior temporal gyrus (ITG.L), and supplementary motor area (SMA.L). **(B)** Patients with T2DM showed decreased ReHo values in the left cerebellum, joint areas of INS/IFG/ROL.L, LING/PCG/PCUN.L, FFG/ITG/PHG.L and increased ReHo value in the bilateral inferior cerebellum; **(C)** Patients with T2DM showed decreased VMHC in CAU, DCG, PreCG, and SMA. Color scale denotes the *t*-value. The *z*-value is the Montreal Neurological Institute (MNI) coordinate.

Detailed group differences are listed in Table 2. For the ALFF analysis, several brain areas showed reduced neural activity in patients with T2DM compared to control subjects, including the surrounding areas of the left inferior temporal gyrus (ITG.L) and the left supplementary motor area (SMA.L). Meanwhile, increased ALFF was detected in the bilateral inferior cerebellum cortex. The spatial distribution of differential brain areas is presented in Figure 1A.

**Table 2:** Differences in amplitude of low-frequency fluctuation (ALFF), regional homogeneity (ReHo), and voxel-mirrored homotopic connectivity (VMHC) values between the patient and control groups [*p* < 0.05, Gaussian Random Field (GRF) corrected].

**No. of voxel cluster**	**Brain regions**	**BA**	**Peak MNI (mm)**			**Peak *t* value**	**Cluster (voxels)**
			***X***	***Y***	***Z***		
**ALFF**							
1	R inferior cerebellum	–	42	−60	−57	5.4421	83
2	L inferior cerebellum	–	−18	−51	−57	4.6161	37
3	L ITG	20/21/28	−48	−3	−33	−4.9119	46
4	L SMA	6	−6	−6	66	−5.1404	57
**ReHo**							
5	R inferior cerebellum	–	24	−33	−45	5.5709	662
6	L inferior cerebellum	–	−21	−39	−54	5.4221	522
7	L FFG/ITG/PHG	20/36	−27	−3	−36	−5.3844	93
8	L LING/CAL/PCUN/PCG	17/18/19/29/30	−27	−57	−6	−4.8231	226
9	L insula/IFG/ROL	13/48	−33	15	−6	−5.6625	257
10	L superior cerebellum	–	−6	−63	−12	−4.4221	78
**VMHC**							
11/12	CAU	–	±15	24	18	−5.0653	40
13/14	DCG	24	±9	0	36	−4.1855	20
15/16	PreCG	6	±36	−6	63	−4.1924	23
17/18	SMA	6	±18	0	57	−4.8424	23

Comparisons were performed at p < 0.05, corrected for multiple comparisons with the GRF method.

IFG, inferior frontal gyrus; ITG, inferior temporal gyrus; SMA, supplement motor area; LING, lingual gyrus; CAL, calcarine fissure and surrounding cortex; DCG, median cingulate and paracingulate gyri; PCUN, precuneus; PCG, posterior cingulate gyrus; FFG, fusiform gyrus; ROL, rolandic operculum; CAU, caudate nucleus; PHG, parahippocampal gyrus; PreCG, precental gyrus; BA, brodmann area; R, right; L, left; x, y, and z coordinates of the primary peak locations in the MNI space; MNI: Montreal Neurological Institute; Negative t-values: patients with type 2 diabetes mellitus (T2DM) < control subjects; Positive t-values: patients with T2DM > control subjects.

A significant decrease of ReHo values in the T2DM group was observed in several brain areas, referring to the left fusiform gyrus (FFG.L), the left inferior temporal gyrus (ITG.L), the left parahippocampal gyrus (PHG.L), the left lingual gyrus (LING.L), the left calcarine cortex (CAL.L), the left precuneus, the insula, the left inferior frontal gyrus (IFG.L), the left rolandic operculum (ROL.L), the left posterior cingulate gyrus (PCG.L), and the superior cerebellum cortex (Figure 1B). However, increased ReHo was found in bilateral inferior semi-lunar lobules of the posterior cerebellum (Figure 1B).

Changes in VMHC were presented in concentrated brain areas involving the caudate, the median cingulate gyrus (DCG), the precentral gyrus (PreCG), and the SMA (Figure 1C).

Amplitude of low-frequency fluctuation and ReHo showed a consistent trend in the bilateral cerebellum posterior lobes, PHG.L, and OTG.L. Meanwhile, ALFF and VMHC exhibited a similar reduction in SMA.L.

### Correlation between Rs-fMRI measurements

An overall description of the correlation is presented in Table 3. Positive correlations were detected in most ROIs, even after a multi-comparison correction. The VMHC-ReHo and ALFF-ReHo group had more common relationships compared to VMHC-ALFF, and the maximum value of correlation coefficients up to 0.79 was obtained by correlating ALFF-ReHo in the right inferior cerebellum. While correlating ALFF and ReHo, the correlation was only missed in SMA.L. The correlation was not significant in the bilateral inferior cerebellum identified by the ALFF method. However, a significant correlation was found in the bilateral inferior cerebellum identified by the ReHo method. The correlation between VMHC and ALFF was less significant and weaker. Several correlation coefficients were preserved only after a multi-comparison correction, including bilateral DCG, PreCG, and the inferior cerebellum identified by the ALFF method.

**Table 3:** Correlation coefficients among ALFF, ReHo, and VMHC in the brain regions showing significant differences.

**No. of cluster**	**VMHC-ALFF**		**VMHC-ReHo**		**ALFF-ReHo**	
	***Corr***	***p*-Value**	***Corr***	***p-*Value**	***Corr***	***p*-Value**
1	0.37	< 0.01^*^	–	0.38	0.60	< 0.01^*^
2	0.41	< 0.01^*^	–	0.35	0.65	< 0.01^*^
3	0.31	< 0.05	0.52	< 0.01^*^	0.67	< 0.01^*^
4	–	0.13	0.58	< 0.01^*^	–	0.23
5	–	0.08	−0.37	< 0.05^*^	0.79	< 0.01^*^
6	–	0.14	−0.28	< 0.05^*^	0.74	< 0.01^*^
7	0.35	< 0.05	0.53	< 0.01^*^	0.66	< 0.01^*^
8	0.32	< 0.05	0.40	< 0.05^*^	0.39	< 0.05^*^
9	0.24	< 0.05	0.36	< 0.05	0.59	< 0.01^*^
10	0.35	< 0.05	0.41	< 0.05^*^	0.37	< 0.05^*^
11	–	0.21	0.55	< 0.01^*^	0.70	< 0.01^*^
12	–	0.13	0.56	< 0.01^*^	0.69	< 0.01^*^
13	0.50	< 0.01^*^	0.73	< 0.01^*^	0.59	< 0.01^*^
14	0.39	< 0.01^*^	0.63	< 0.01^*^	0.54	< 0.01^*^
15	0.49	< 0.01^*^	0.59	< 0.01^*^	0.54	< 0.01^*^
16	0.37	< 0.05^*^	0.52	< 0.01^*^	0.55	< 0.01^*^
17	–	0.42	0.40	< 0.05^*^	–	0.12
18	–	0.61	0.43	< 0.01^*^	0.28	< 0.05

^*^Denotes a significant p-value after a multi-comparison correction. Corr denotes Pearson's correlation coefficient.

The number index of the cluster corresponds to Table 2.

### Correlation with clinical variables

**Figure 2:**
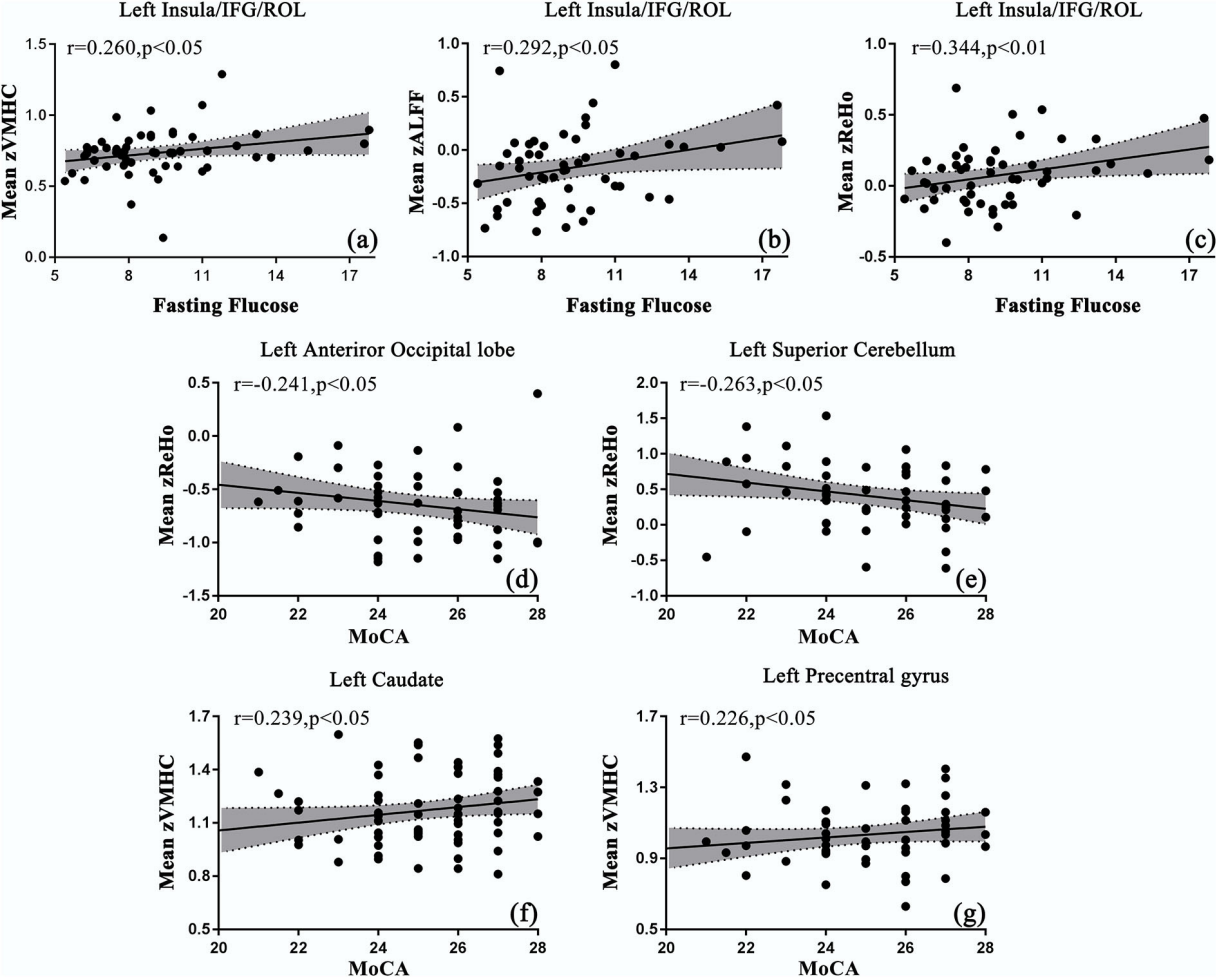
Correlations between the mean values of resting-state functional magnetic resonance imaging (rs-fMRI) indices and clinical parameters in patients with T2DM. **(a–c)** The mean values of ALFF/ReHo/VMHC were positively correlated with fasting glucose in s INS/IFG/ROL.L; **(d, e)** the mean values of ReHo were negatively correlated with Montreal Cognitive Assessment (MoCA) scores in the left occipital lobe and cerebellum; **(f, g)** the mean values of VMHC were positively correlated with the MoCA scores in CAU.L and PreCG.L.

The disease duration was positively correlated with the ALFF values in the right SMA (SMA.R), and ReHo values in the bilateral caudate, PreCG, and SMA.R. BMI was positively correlated with the ReHo values in the bilateral DCG and negatively correlated with the ALFF values in the bilateral inferior cerebellum (Table 4). As shown in Figure 2, the mean values of ALFF/ReHo/VMHC were all positively related to fasting glucose in the joint areas of INS/IFG/ROL.L. Negative correlations between MoCA scores and mean ReHo values were found in the left anterior occipital lobe and the superior cerebellum cortex. Positive correlations were also found between MoCA scores and mean VMHC values in CAU.L and PreCG.L. However, no correlation was preserved after a multi-comparison correction.

**Table 4:** Correlations between clinical parameters and the mean values of resting-state functional magnetic resonance imaging (rs-fMRI) indices extracted from significantly different brain regions.

**Region**	**Pair**	***Corr***	***p*-Value**
L CAU	ReHo-duration	0.325	< 0.005
R CAU	ReHo-duration	0.265	< 0.05
L DCG	ReHo-BMI	0.306	< 0.05
R DCG	ReHo-BMI	0.239	< 0.05
R SMA	ALFF-duration	0.378	< 0.005
	ReHo-duration	0.245	< 0.05
L PreCG	ReHo-duration	0.243	< 0.05
R PreCG	ReHo-duration	0.273	< 0.05
L inferior cerebellum	ALFF-BMI	−0.377	< 0.005
R inferior cerebellum	ALFF-BMI	−0.310	< 0.01

The number index of the cluster corresponds to Table 2.

Corr denotes Pearson's correlation coefficient.

### Mediation analysis

Exploratory mediation analyses were performed. However, no significant indirect effects were observed between FPG/duration of disease and the MoCA score with the identified abnormal brain activity as a mediator (corrected with age and sex).

## Discussion

In this study, we identified significant intrinsic brain activity alterations in patients with T2DM compared to healthy controls from various aspects. Patients with T2DM showed the coexistence of increased ALFF and ReHo in the bilateral inferior cerebellum, decreased ALFF and VMHC in SMA.L, and decreased ALFF and ReHo in ITG.L. Other neural abnormalities of decreased brain activity were found in brain areas involving visual, motor, and sensory processing using a single method. ReHo was commonly correlated with ALFF and VMHC in brain regions exhibiting differences. Meanwhile, a correlation was identified between fasting glucose and ALFF/ReHo/VMHC in the surrounding areas of INS.L. ReHo represented neural abnormalities in the left anterior occipital lobe and the superior cerebellum, and VMHC in CAU.L and PreCG.L was correlated with the cognitive performance of patients.

Amplitude of low-frequency fluctuation, ReHo, and VMHC are calculated based on different neurological mechanisms and have been used to investigate the neuropathology of various mental disorders (Cui et al., 2014; Li et al., 2021; Liu, J. et al., 2021). ALFF denotes a higher level of regional neural activity with increased metabolic rates of oxygen and glucose (Liu et al., 2016). ReHo demonstrates the neural coherence of a single voxel with its neighbors. While healthy brains are equipped with a specific pattern of synchronies between the two hemispheres, VMHC can be used as a biomarker for measuring neural activity. In this study, the coexisting abnormalities of rs-fMRI indices in brain regions may represent more severe functional changes than those reflected in a single method. In addition, more reliable information has been provided, which is necessary for understanding the mechanism of cognitive impairment in patients with T2DM. However, combining the results of three rs-fMRI indices can provide a wide range of abnormal brain regions than a single method, making full use of each index to achieve complementary results. Based on this, we identified more regions by putting ALFF, ReHo, and VMHC results together in the current study. Synthesizing the aforementioned information may provide a feasible way to explore the central question of how the brain's regional function is manifested in T2DM beyond a meta-analysis (Liu, J. et al., 2021).

In the current study, the cerebellum was found to show altered spontaneous neural activity. A significant increase in spontaneous neural activity was observed in the inferior cerebellum. Convergent evidence demonstrated that the cerebellum plays a vital role in cognitive, emotional, and sensory processing. Xia et al. (2013) interpreted increased neural activity in the cerebellum as compensation for the loss of cognitive function in other brain areas. This may be part of the reason why MoCA scores in patients with T2DM show no differences compared to healthy controls (Cui et al., 2014; Wang et al., 2017; Qi et al., 2020). In addition, cerebellum dysfunction was considered the secondary symptom of microvascular complications, such as diabetic retinopathy or nephropathy (Tong et al., 2014). However, patients with T2DM in the current study did not have clinical retinopathy. Hence, cerebellar dysfunction may be an early change before the advanced stage of visual changes. Bai et al. (2011) reported increased ALFF in the cerebellar posterior lobe in patients with mild cognitive impairment. This may provide further indication of an increased risk for dementia in patients with T2DM. Decreased ReHo was also found in a smaller region in the superior cerebellum. Several studies reported decreased neural activity in the cerebellum (Peng et al., 2016; Wang et al., 2017; Liao et al., 2019; Shi et al., 2020; Zhang et al., 2020). However, all patients enrolled in these studies had diabetic complications. We speculated that there was a decrease in activity ahead of a clinical lesion.

Decreased spontaneous neural activity was found only in the left hemisphere of the cerebrum. The joint areas surrounding SMA.L, ITG.L, INS.L, and the anterior occipital lobe mainly involve working memory, emotional cognition, motor function, and sensory processing. The SMA area is involved in a sequential movement generated from memory and cognition control *via* indirect ways. Researchers reported that the loss of motor function might share a common cause with cognitive decline (Buchman and Bennett, 2011; Cañas et al., 2018). ITG.L presented an inconsistent trend compared to previous studies, where increased ALFF in this brain area was reported (Wang et al., 2019). Researchers considered that increased ALFF values in ITG.L might be the effects of compensation mechanisms in a visual network or a default mode network (Wang et al., 2019; Qi et al., 2020). While ITG played an important role in mediating visual function, the reduction of rs-fMRI indices could be an early marker of sensory dysfunction before clinical symptoms. The surrounding areas of the insula are responsible for basic functions of cognition and bodily awareness, for example, pain perception. An indirect linkage between the insula and obesity was claimed by Steward et al. (2016), thus the insula may be involved in the development of T2DM. In addition, the hemispheric dominance of the left insula in emotion and perception was reported (Kann et al., 2016), making our consistent finding more reliable. Areas in the anterior occipital lobe, including PCG, LING, PCUN, and CAL, play an important role in information transfer and have been implicated in some neuropsychiatric diseases including impairment or dementia (Ebert and Ebmeier, 1996; Aminoff et al., 2013; Tan et al., 2013; Liu, H. et al., 2021). A previous rs-fMRI study suggested that ALFF was enhanced in patients with AD located in PHG.L, ITG.L, SMA, FFG.L, and the cerebellum, while it was reduced in several other regions (Wang et al., 2011). In our study, a significant hypoactivity was found in these brain areas. While T2DM is a known risk factor for dementia, further studies are needed to build a more direct relation between T2DM and the development of dementia.

Significant correlations were found in the correlation analysis between rs-fMRI indices in the identified brain ROIs. Notably, the correlations went unnoticed in a previous study. For instance, abnormalities were detected in the insula area only by the ReHo method, but VMHC and ALFF were significantly correlated with ReHo in the insula. Cui et al. (2014) only demonstrated a positive correlation between ALFF and ReHo in the occipital lobe showing coexisting functional intensity and coherent abnormalities. Hu et al. (2021) only compared the differences in rs-fMRI metrics distributed over the brain. Our results indicate that coexisting abnormalities were popular even in brain areas not identified by various rs-fMRI indices at the same time.

Correlation analyses were conducted to investigate the relationship between clinical variables, rs-fMRI indices, and cognition. Fasting glucose and MoCA were found to be significantly correlated with rs-fMRI indices in several brain regions. Notably, fasting glucose was positively correlated with ALFF, ReHo, and VMHC in the surrounding areas of the insula at the same time. A strong relationship between higher blood sugar levels and abnormalities in the surrounding areas of the insula indicated a vulnerable target in the brain for T2DM. Decreased neural activity in the surrounding areas of the insula may act as an early biomarker of its potential contribution to cognitive decline. However, Cui et al. (2014) found no correlation between blood glucose and changes in neural activity and considered the blood as a contributor to the difference in neural activity. Therefore, further studies will be needed to test the relationship. The MoCA score was negatively correlated with neural abnormalities in the anterior occipital lobe and superior cerebellum. Xia et al. (2015) interpreted the relationship as a compensatory mechanism for the loss of cognitive function. Xiong et al. (2020) further inferred that more severely impaired cognition may arouse enhanced alterations in the brain regions. Meanwhile, MoCA was positively correlated with VMHC in the left caudate and the left precentral gyrus. These results were likely to imply a sequential occurrence of neural abnormalities along with changes in MoCA. Considering that MoCA scores showed no between-group differences, it will be interesting to follow these patients over time to determine the clinical significance of these findings. The problem is that no correlation was preserved after a multi-comparison correction. Further verification will be needed in the following study.

Additionally, we performed a mediation analysis to further investigate the mechanism of the occurrence of cognitive impairment. Mediation is a natural fit for neuroimaging data due to its capability to explore the relationship between experimental variables and outcomes in a single model. Researchers quantified the mediation effect between clinical status and psychiatric symptoms through brain activity/structure (Cheng et al., 2018, 2021). Zhang et al. (2018) conducted a mediation analysis to determine whether the olfactory system acted as a mediator between diabetic parameters and cognitive function. The study found that olfactory function mediated the relationship between a diabetic parameter and an executive function, indicating a pathway throughout insulin, olfactory function, and cognitive impairment. In contrast, our study revealed no significant indirect effect. In the future, we will conduct an in-depth analysis *via* reviewing the methodology of rs-fMRI indices again and the regrouping of participants more strictly to clarify the underlying principle.

Our study has several limitations. First, medication or treatment received by patients may affect neural activity. For example, hypoglycemia is a common side effect in medication treatment for diabetes (Seaquist et al., 2013). It has been reported that hypoglycemia induces adaptive changes in brain metabolism (Rehni and Dave, 2018). Although we attempted to exclude patients with a history of severe hypoglycemic episodes as far as possible, there is still a possible interference caused by hypoglycemia. In case of possibility, medication-naive patients with T2DM will be specifically included to remove this bias. Second, disease duration may be another factor related to cognitive function. Reinke et al. (2022) reported a U-shaped association of T2DM duration and the risk of dementia. A detailed group design based on different periods of disease duration would help to interpret the neural activity patterns. Third, neuropsychological tests performed in this study are not comprehensive enough. More tests may be helpful to correlate brain alterations with clinical manifestations. Finally, our study is still a cross-sectional design. van den Berg et al. (2010) conducted a 4-year observation to follow the evolution of cognitive decrements in patients with T2DM. Therefore, a longitudinal MRI study will enable us to investigate the dynamic changes in brain function in the development of T2DM.

## Conclusion

In conclusion, the combination analyses of ALFF, ReHo, and VMHC demonstrated a wide range of abnormal spontaneous neural activity in brain regions, which were mainly involved in motor function and sensory processing and indirectly involved in cognitive function. Meanwhile, the indices were correlated commonly in the detected brain areas, further demonstrating the need for combination analyses. The fasting glucose level and MoCA score were significantly correlated with the indices in the left insula and the motor/visual-related regions. These findings emphasize the importance of glycemic control in preventing functional abnormalities in the brain and caution patients with T2DM to pay more attention to motor/visual function to monitor for early cognitive decline. This study provides a new approach to exploring abnormal neural activity in patients with T2DM and enhances our understanding of a neuromechanism linking T2DM and cognitive impairment.

## Data availability statement

The raw data supporting the conclusions of this article will be made available by the authors, without undue reservation.

## Ethics statement

The studies involving human participants were reviewed and approved by Research Ethics Committee (REC) of Henan Provincial People's Hospital. The patients/participants provided their written informed consent to participate in this study.

## Author contributions

GZ and TL: data analyzing and manuscript writing. WW and RZ: data collection. HW and MW: experiment design. All authors contributed to the article and approved the submitted version.
